# Increased left inferior fronto-striatal activation during error monitoring after fMRI neurofeedback of right inferior frontal cortex in adolescents with attention deficit hyperactivity disorder

**DOI:** 10.1016/j.nicl.2020.102311

**Published:** 2020-06-10

**Authors:** M. Criaud, M. Wulff, A.A. Alegria, G.J. Barker, V. Giampietro, K. Rubia

**Affiliations:** a Department of Child and Adolescent Psychiatry, Institute of Psychiatry, Psychology and Neuroscience, King’s College London, London, UK; b Department of Neuroimaging, Institute of Psychiatry, Psychology & Neuroscience, King’s College London, London, UK

**Keywords:** ADHD, fMRI, Neurofeedback, Error monitoring

## Abstract

Attention Deficit/Hyperactivity Disorder (ADHD) is a self-regulation disorder, with impairments in error monitoring associated with underactivation of the related brain network(s). Psychostimulant medication improves ADHD symptoms and can upregulate brain function, but has side effects, with limited evidence for longer-term effects. Real-time functional magnetic resonance neurofeedback (fMRI-NF) has potential longer-term neuroplastic effects. We previously reported the effects of 11 runs of 8.5 min of fMRI-NF of the right inferior frontal cortex (rIFC) in adolescents with ADHD. This resulted in improvement of clinical symptom and enhanced rIFC activation post-pre treatment during response inhibition, when compared to a control group receiving fMRI-NF of the left parahippocampal gyrus (lPHG).

In the current study we applied a novel analysis to the existing data by investigating the effects of fMRI-NF of rIFC in 16 adolescents with ADHD compared to fMRI-NF of lPHG in 11 adolescents with ADHD on the neurofunctional correlates of error monitoring during the same fMRI tracking stop task and potential associations with cognitive and clinical measures. We found stronger performance adjustment to errors in the rIFC-NF compared to the control lPHG-NF group. At the brain function level, fMRI-NF of rIFC compared to that of lPHG was associated with increased activation in error monitoring regions of the left IFC, premotor cortex, insula and putamen. The increased activation in left IFC-insular-striatal error monitoring regions in the rIFC-NF relative to the lPHG-NF group was furthermore trend-wise correlated with NF-induced ADHD symptom improvements.

The findings of this study show, that during error monitoring, fMRI-NF training of rIFC upregulation elicited improvement in post-error behavioural adjustments and concomitant increased activation in left hemispheric fronto-insular-striatal and premotor regions mediating self-control and self-monitoring functions. This suggests that the administration of fMRI-NF of the rIFC may have had an impact on wider networks of self-regulation and self-monitoring in adolescents with ADHD.

## Highlights

• ADHD patients have performance and brain activation deficits during error monitoring.
• fMRI-Neurofeedback of right inferior frontal cortex improved error monitoring in ADHD.
• It increased left fronto-striatal error monitoring activation in ADHD.
• fMRI Neurofeedback improves performance and activation of error monitoring in ADHD.

## 1 Introduction

Attention Deficit/Hyperactivity Disorder (ADHD) is a childhood disorder characterised by a persistent pattern of age-inappropriate and impairing problems with inattention and/or impulsiveness/hyperactivity (American Psychiatric Association, 2013). ADHD has been described as a self-regulation disorder, with impairments in cognitive control functions such as inhibitory control and error monitoring (Geburek et al., 2013, Groom et al., 2013, Mohamed et al., 2016a, Rubia et al., 2001, Rubia et al., 2007a, Shiels and Hawk, 2010). Inhibitory control refers to the ability to refrain a behaviour (Munakata et al., 2011), and is typically measured in Go/No-go or Stop tasks. In the former, a motor response to frequent Go signals, triggering prepotent response tendencies, has to be selectively inhibited after the appearance of a less frequent No-go signal, while in the latter the response must be withdrawn after the appearance of a low frequency Stop signal (Rubia et al., 2007a, Verbruggen et al., 2019). Motor inhibitory control has been associated most prominently with the activity of the right inferior frontal cortex (IFC), dorsolateral prefrontal cortex (DLPFC), pre-supplementary motor area (pre-SMA), striatum and subthalamic nucleus in children and adults (Criaud and Boulinguez, 2013, Munakata et al., 2011, Rubia et al., 2001, Rubia et al., 2003b, Rubia et al., 2006, Rubia et al., 2007a, Zandbelt and Vink, 2010). Error monitoring refers to the ability to adjust a behaviour after an error. It has been shown to be mediated by the anterior cingulate cortex (ACC), mesial and middle frontal cortex, SMA, bilateral IFC, anterior insula, the putamen and the caudate (Chevrier and Schachar, 2010, Chevrier et al., 2007, Costa et al., 2013, King et al., 2010, Li et al., 2008, Rubia et al., 2003b, Schroder and Moser, 2014, Sharp et al., 2010, Ullsperger and von Cramon, 2006, Zhang and Li, 2012, Zhang et al., 2017).

Patients with ADHD have been shown to have consistent impairments in inhibition and error monitoring (Lipszyc and Schachar, 2010, Rubia et al., 2001, Rubia et al., 2007a, Shiels and Hawk, 2010, Willcutt, 2018). Individual and meta-analytic studies have consistently shown reduced activation in the right IFC/anterior insula, striatum, ACC and pre-SMA in children and adults with ADHD during tasks of motor and interference inhibition (Hart et al., 2013, Hart et al., 2014, Norman et al., 2016, Rubia et al., 2005, Rubia et al., 2008). During error monitoring, medication naïve patients with ADHD have shown underactivation of IFC and DLPFC, premotor, pre-SMA, superior and inferior parietal cortices, posterior cingulate (PCC)/precuneus, posterior thalamus and inferior temporo-occipital areas (Chantiluke et al., 2015, Rubia et al., 2005, Rubia et al., 2011b). Adults with a history of ADHD also present decreased activation in the right IFC, anterior insula, striatum and bilateral thalamus (Cubillo et al., 2010). The neural correlates of error monitoring have furthermore been found to be correlated with the severity of hyperactivity and impulsivity symptoms in adults with ADHD (Balogh et al., 2017).

Psychostimulant medication is the gold-standard treatment for ADHD. Stimulants are associated with improvement of clinical symptoms in about 70% of patients (Cortese et al., 2018, Sudre et al., 2018). Stimulants, most prominently Methylphenidate (MPH), have also been shown to have an ameliorating effect on cognitive (Bédard et al., 2015, Coghill et al., 2014, Fredriksen et al., 2019, Hammerness et al., 2014) and brain function impairments in ADHD (Rubia et al., 2014a). Our meta-analysis of stimulant effects on ADHD brain function in fMRI studies showed increased activation relative to placebo or off medication in the right IFC, anterior insula, and (at a more lenient statistical threshold) in the putamen (Rubia et al., 2014b). Stimulants have also been shown to downregulate the dorsomedial prefrontal cortex, presumably reducing enhanced activation in an area of the default mode network (DMN) (Rubia et al., 2014b). Furthermore, several individual studies have shown that stimulant medication can improve and/or normalise brain underactivation in key frontal and striatal regions during response inhibition (Cubillo et al., 2014, Rubia et al., 2011b, Rubia et al., 2014b) and other cognitive functions (Bush et al., 2008, Rubia et al., 2009a, Rubia et al., 2009b).

MPH has also been shown to influence error monitoring networks in children with ADHD. Thus a single dose of MPH resulted in a complete normalisation of the brain underactivation in children with ADHD during placebo in error monitoring regions compared to healthy controls, i.e. in bilateral IFC, insula, putamen, right caudate, left medial frontal cortex, parietal and occipital regions (Rubia et al., 2011b).

Although stimulants are effective in treating ADHD symptoms, they have important limitations such as the potential for side effects, and the fact that they are not indicated for all patients (Cortese et al., 2018, Cunill et al., 2016, Molina et al., 2009, Storebø et al., 2015, Swanson et al., 2018, Zuddas et al., 2018). In addition, their long-term efficacy has been questioned (Cortese et al., 2018, Molina et al., 2009, Swanson et al., 2018, Zuddas et al., 2018) which may be linked to evidence for brain adaptation to the drug, as shown in positron emission tomography studies (Fusar-Poli et al., 2012, Wang et al., 2013). Also, the long-term effects of these drugs on the development of brain structure or function is relatively unknown. Therefore, alternative treatments with potential longer-term efficacy are highly desirable. One potential treatment that has been shown to have longer-term neuroplastic effects is neurofeedback (NF). EEG-NF has been tested in ADHD for over 50 years, but recent meta-analyses (Cortese et al., 2016, Thibault et al., 2015) have shown limited efficacy. fMRI-NF has superior spatial resolution to EEG-NF and can be used to teach participants to self-regulate the blood-oxygen level-dependent (BOLD) response in specific brain regions or networks that are dysfunctional in the disorder. Real-time feedback of participants’ own brain activity can be presented as a computer game to improve engagement. fMRI-NF is a very promising novel neurotherapy in clinical populations such as ADHD, as it can target impaired brain regions and has no known side effects (Rubia, 2018).

A recent randomised controlled trial from our lab tested fMRI-NF of the rIFC compared to fMRI-NF of the left parahippocampal gyrus (lPHG) in adolescents with ADHD (Alegria et al., 2017, Rubia et al., 2019). Thirty-one boys with a clinical ADHD diagnosis underwent 11 runs of 8.5 min of fMRI-NF during 4 1-1.15 hour long scans over a 2-week period, with a rocket movie as feedback. Eighteen participants learned to self-upregulate the target region, the rIFC (rIFC-NF group); while 13 participants self-upregulated a control region, the lPHG (lPHG-NF group). In both groups activation of their target regions increased linearly across the 11 fMRI-NF runs. However, only the rIFC-NF group showed a transfer effect (self-regulation without feedback, as a proxy of transfer to real life) that significantly correlated with reduced ADHD symptoms. Although ADHD symptoms significantly improved in both groups, only the rIFC-NF group showed a large reduction of symptoms at 11 months follow-up, with an effect size of almost 1, compared to a trend-level reduction in the lPHG-NF group. The rIFC-NF group also showed trend-level improved sustained attention performance. In addition to the linear increase of activation of the rIFC in the rIFC-NF group, there was an increase in functional connectivity between the rIFC and the ACC and caudate, and a decrease in functional connectivity between the rIFC and regions of the posterior default mode network (DMN). This suggested that the NF of an isolated region led to positive network changes in cognitive control and DMN networks (Rubia et al., 2019). In order to measure the effects of fMRI-NF on brain function in ADHD, the participants of this study also performed a motor response inhibition fMRI task, the tracking stop signal task, before and after fMRI-NF. The tracking stop task dynamically adjusts the timing of the stop signal in order that all participants fail on 50% of the trials and hence measures brain response to both successful and failed inhibition. Comparing results post minus pre fMRI-NF, during successful inhibition there was increased activation of the rIFC and parietal regions in the rIFC-NF group relative to the lPHG-NF group (Alegria et al., 2017). Similar upregulation and normalisation effects have been observed in the same region when comparing the effect of stimulant medication relative to placebo, using the same stop task (Cubillo et al., 2014, Rubia et al., 2011b, Rubia et al., 2014b). This suggests that fMRI-NF of the rIFC has similar brain activation effects on the disorder as stimulant medication, but without side effects.

In our previous work, we did not, however, investigate the effects of fMRI-NF on the 50% of *failed* stop trials, which measure error monitoring. The aim of the current study was therefore to investigate whether fMRI-NF of rIFC also improved brain activation related to error monitoring in the stop task, in the same group of adolescents with ADHD (Alegria et al., 2017). The use of the tracking stop task makes it possible to investigate the effects of fMRI-NF of rIFC on the 50% unsuccessful stop trials pre and post the fMRI-NF intervention. We also assessed whether fMRI-NF related changes were associated with changes in clinical and neuropsychological measures. We hypothesised that fMRI-NF of the rIFC compared to fMRI-NF of the lPHG would increase brain activation during failed stop trials in error monitoring regions such as bilateral IFC, insula, striato-thalamic, parietal, temporal, anterior and posterior cingulate. We furthermore hypothesised that these changes would be related to improved error monitoring performance and improvement in clinical symptoms.

## 2 Materials and methods

The study was a single-blind randomised controlled trial investigating the effect of fMRI-NF of the rIFC in 18 boys with ADHD (rIFC-NF group) compared to an active control group of 13 boys with ADHD (lPHG-NF group). For the purpose of this analysis, only the participants who completed the stop task were included, resulting in 16 in the rIFC-NF group and 11 in the lPHG-NF group. The experimental design is detailed in (Alegria et al., 2017).

### 2.1 Participants

Twenty-seven boys with ADHD between 12 and 17 years of age (mean (SD) = 14 (1.5)) were included in this study. The diagnosis of ADHD was made by an experienced child psychiatrist and confirmed with Schedule of Affective Disorders and Schizophrenia for School-Age Children-Present and Lifetime version (K-SADS-PL) (Kaufman et al., 1996). Twenty-four participants met the DSM-5 criteria for the ADHD combined hyperactive/impulsive subtype, while three met the criteria for the ADHD inattentive subtype. They also had to score above the clinical ADHD threshold on the Conners’ Parent Rating Scale (CPRS-R), a parent rated index of ADHD severity (Conners et al., 1998). The Social Communication Questionnaire (SCQ) (Rutter et al., 2003) was used to screen for autism spectrum disorders. Two boys met/exceeded the cut-off score of 15, but in both cases a potential autism spectrum condition was ruled out by clinical interview. The Children’s Global Assessment Scale (CGAS) (Shaffer et al., 1983) was also used to assess for general functioning and symptom severity.

Exclusion criteria were IQ below 80 as measured on the Wechsler Abbreviated Scale of Intelligence (WASI; Wechsler, 1999), alcohol and substance abuse, neurological and comorbid psychiatric disorders (except for disruptive behaviour disorder), and MRI contraindications. Fourteen rIFC-NF participants and seven participants in the lPHG-NF group received psychostimulant medication during the study (rIFC-NF group: methylphenidate: N = 12, dexamphetamine: N = 2; lPHG-NF group: methylphenidate: N = 7); in all cases the medication regime was stable, with a period of at least seven days after titration before testing started. One boy in the lPHG-NF group was medication-naïve. Two participants in the rIFC-NF group and three in the lPHG-NF group stopped taking their ADHD medication at least seven days before their participation in the study. A chi-square test was used to test the difference in medication status between the two groups.

The local research ethics committee approved the study, which was conducted in accordance with the Declaration of Helsinki (Research Ethics Committee reference number; 12/LO/0708). Each participant/legal guardian gave informed written consent/assent. All participants received up to £150 for their participation to the study: £20 for each fMRI-NF scan, up to £10 for best performance during the session, as well as £30 for the post-training visit. They were all reimbursed for their travel expenses.

### 2.2 Clinical outcome measures

Both primary and secondary outcome measures were parent-rated questionnaires, the ADHD rating scale (ADHD-RS) and the CPRS-R, respectively. The ADHD-RS assesses ADHD symptoms according to the DSM-5 and also monitors treatment effects (Dupaul et al., 1998). The CPRS-R was used to record the frequency of symptoms in the everyday lives of patients (Conners et al., 1998). The Weekly Parent Ratings of Evening and Morning Behavior-Revised scale (WREMB-R, (Wehmeier et al., 2009)) and the Columbia Impairment Scale-Parent version (CIS, (Bird et al., 1993)) were used to measure ADHD-related difficulties and functional impairment, respectively.

### 2.3 FMRI-NF protocol

The fMRI-NF protocol consisted of four 1 h to 1.15 hr sessions within a 2-week period, totalling a maximum of 14 NF runs of 8.5 min each. Each fMRI-NF run consisted of 6 activation blocks of 40 s, separated by a rest block of 30 s. The run always started with a rest block showing an image of a dolphin. In the activation blocks, the neurofeedback of the target region was represented by a video of a rocket, which the participants were asked to move toward space using any strategy they could. Since explicit instructions have been shown to be less effective (Sulzer et al., 2013) and instruction-free NF has been recommended for EEG-NF in children with ADHD (Gevensleben et al., 2014, Strehl et al., 2017), minimal instructions were given to participants. The video of the rocket displayed a continuous feedback, meaning that the direction and distance travelled in space was updated every MRI repetition time (TR, i.e., 2 s), based on the change in BOLD response in the region of interest (ROI) compare to that of a control region (white matter). To increase the participants’ motivation, a score (0–10), based on the distance travelled during each run, was calculated and displayed on the screen after each activation/training block. The participants received a monetary reward for their best performance in the session. The fMRI-NF performance was acquired for each run, for each participant as another way to measure brain regulation capacity.

In addition to the fMRI-NF sessions, participants were instructed to practice daily brain self-regulation using a picture of the rocket. After the last fMRI-NF run, the participants underwent a “transfer” run, where they tried to self-regulate their brain without any feedback being presented. This task measures retention of learning and transfer of training strategies to everyday life (Alegria et al., 2017, Drechsler et al., 2007).

### 2.4 FMRI stop task

Before and after NF training, participants underwent an fMRI tracking stop task (Rubia et al., 2003b, Rubia et al., 2005, Rubia et al., 2011a, Rubia et al., 2011b, Rubia et al., 2014b). The participants practiced the stop task once prior to the scan. The tracking stop task is a very efficient tool to investigate motor response inhibition as well as error monitoring. It is a choice reaction time task where participants are instructed to respond as fast as they can to arrows pointing left or right. In 20% of the trials, this “go” signal is followed by a stop signal, and participants have to inhibit their response. The task consists of 156 go trials, with a mean intertrial-interval of 1.8 s and 40 stop signals, pseudo-randomly distributed, appearing about 250 ms after the go signal. A tracking algorithm changes the time interval between go signal and stop signal onsets according to each subject’s inhibitory performance, to ensure that the task is equally challenging for each individual and to result in 50% successful and 50% unsuccessful inhibition trials in each run.

The contrast of motor response inhibition measures in the 50% *successful* stop versus go trials has been published previously (Alegria et al., 2017). In the current study, we focussed instead on the contrast of *unsuccessful* stop trials minus go trials which measures the brain response that mediates error monitoring (Rubia et al., 2003b, Rubia et al., 2011b). The key dependent variable of interest for this analysis was therefore the post-error reaction time to go signals, i.e. the reaction time to a go stimulus immediately after a stop failure, as this variable reflects the cognitive adjustment to making an error (Rubia et al., 2011b). Other variables assessed were the dependent variable for the inhibitory process: the stop signal reaction time (SSRT), which is calculated by subtracting the mean stop signal delay from the mean reaction time to go trials (Logan et al., 2014); the mean reaction time to go signals, which reflects the go process of the task; the standard deviation of reaction times to go signals, which represents intrasubject reaction time variability; and omission errors to go signals, which are related to inattention.

### 2.5 FMRI data acquisition and processing

All participants who completed the stop task (16 participants in the rIFC-NF group and 11 in the lPHG-NF group) were included in the analyses. Full details of the fMRI-NF data acquisition and processing are available in Alegria et al. (2017) and in the supplementary material.

A custom fMRI-NF interface system (Bodurka and Bandettini, 2008) and the AFNI software (Cox, 1996) were used for real-time transfer and analysis of the fMRI data. Two target ROIs (rIFC and lPHG - ROI_TAR_) were defined in Talairach space and mapped to the individual participant’s fMRI scans via the T1-weighted structural image and a two-volume EPI localizer image collected at the start of each session. In addition, a mask of the white matter was also created for use as reference ROI (ROI_REF_) to compensate for non-specific global brain effect. The mean BOLD signal was extracted from each ROI by applying the masks to the pre-processed fMRI data. For each new brain volume (e.g. every TR/two seconds), AFNI calculated a new set of values for each ROIs. These values were transferred to an in-house program that generated the feedback by displaying a moving rocket. The neurofeedback signal was calculated as:

((ROI_TAR_-ROI_REF_) – (ROI_TAR__Previous_ – ROI_REF__Previous_))

where ROI_TAR__Previous_ and ROI_REF__Previous_ are the average activation of rIFC or lPHG, and of white matter, respectively, during the previous rest block. In other word, the NF signal was a function of the difference current ROI_TAR_ activity (averaged over 3 TR periods, in order to smooth the data) to the averaged activity of the previous rest block. All values were being measured relative to the corresponding white matter signal, that represents global signal of no interest. Before each fMRI-NF run, participants were informed/reminded of the delay (at least 6 s) in the feedback they would receive, caused by both hemodynamic delay and data processing time.

Details of the acquisition of the fMRI Stop task can be found in Alegria et al. (2017) and in the supplementary material.

### 2.6 Data analyses

#### 2.6.1 Behavioural data

Some parents did not complete all questionnaires. Two ADHD-RS and 3 CPRS-R parent ratings were missing across all participants and all visits. There was a limited amount of missing data (<5%) and missing points were assumed to be completely at random. Multiple (i.e., 20) imputations were used for all missing data. The individual estimates from the multiply-imputed datasets were then used to calculate a combined estimate by applying Rubin’s Rules (Little and Rubin, 2019). Repeated-measures mixed ANOVAs were used to test pre- and post-fMRI-NF effects on the clinical outcome measures and the stop task dependent variables, within and between groups. Two-tailed Pearson analyses were used to test for correlations between fMRI-NF induced brain activation changes during failed stop trials (Post-fMRI-NF – Pre-fMRI-NF) and clinical and cognitive changes (Post-fMRI-NF – Pre-fMRI-NF). The behavioural analyses were not corrected for multiple comparisons.

The fMRI-NF performance was averaged across all runs and a between-group ANOVA was conducted to assess group differences in this performance.

#### 2.6.2 FMRI stop task

The individual and group-level analysis methods are described in detail elsewhere (Brammer et al., 1997, Bullmore et al., 1999a, Bullmore et al., 1999b). Version 4.1 of the non-parametric XBAM software package (Brammer et al., 1997) was used to analyse the data. The data analyses details are described in detail in Alegria et al. (2017) and in the supplementary material. The fMRI data underwent realignment, motion correction, smoothing, global detrending and spin-excitation history correction. The time series analysis of individual data was then performed with a wavelet-based data resampling method (Bullmore et al., 1999b, Bullmore et al., 2001).

*Individual Analysis.* The main experimental condition of error monitoring in the stop task, e.g. failed stop trials, against an implicit baseline, e.g. the go trials, was obtained using a standard GLM approach. Individual goodness-of-fit statistic (SSQ-ratio) maps were created and transformed into standard space (Talairach and Tournoux, 1988).

*Group Analysis*. Two group activation maps (pre- and post-fMRI-NF) for each NF group were produced for the experimental condition. A repeated-measures ANOVA was then conducted to test the interaction of time (pre, post) by group (rIFC-NF group, lPHG-NF group). The voxel-level threshold was first set to p < 0.05 to give maximum sensitivity and to minimize type II errors. After that, a cluster-level threshold was computed for the resulting 3D voxel clusters and set in such a way as to produce less than one false positive cluster per map, in this analysis achieved at cluster-level p < 0.02. The cluster p-value is adjusted to eliminate any potential false positive cluster from the map, i.e. we can be confident that all the clusters we see are not false positives (based on our non-parametric data-driven analysis). The necessary combination of voxel- and cluster-level thresholds was not assumed from theory but rather was determined by direct permutation, giving excellent type I error control (Bullmore et al., 1999b). Cluster mass rather than a cluster-extent threshold was used to minimize discrimination against possible small, but strongly responding, foci of activation (Bullmore et al., 1999b).

#### 2.6.3 Group activation across all 11 fMRI-NF runs in both groups

To elucidate which brain regions were activated generically, independent of group, during the fMRI-NF relative to baseline, we conducted a group activation map across all participants (rIFC-NF and lPHG-NF groups) for the NF condition versus baseline, averaged across all 11 runs. For this purpose, a group brain activation map was produced for the fMRI-NF condition contrasted with the baseline condition. Hypothesis testing was carried out at the cluster level. A voxel-wise test at p < 0.05 was conducted to identify any voxels that might plausibly be activated, followed by a subsequent test at a cluster-level threshold of p < 0.011 to remove false positive clusters produced by the voxel level test. Combined voxel/cluster tests with permutation testing allow for excellent Type 1 error control (Bullmore et al, 1999b). For the group activation analysis, < 1 false activated 3D clusters were expected at a p value of < 0.05 at voxel-level and p < 0.001 at cluster-level comparisons.

## 3 Results

### 3.1 Clinical outcome measures

The between-group ANOVA revealed no significant between-group differences at baseline in demographic or clinical measures, in medication type or medication dose (Table 1). The chi-square analysis also showed no significant difference in medication status between the groups (Pearson chi-square = 2.70, p = 0.259). The average fMRI-NF performance was significantly higher in the rIFC-NF group (mean = 57, SD = 8.4) compared to the lPHG-NF group (mean = 43, SD = 10.0) (df = 1,25) = 17.479, p < 0.001).

**Table 1:** Demographic, clinical, and medication status characteristics and number of fMRI-NF runs in active and control ADHD groups at baseline.

	rIFC-NF group (N = 16)	lPHG-NF group (N = 11)	Between-subject ANOVA	
	Mean (SD) or n (%)	Mean (SD) or n (%)	F (1,25)/χ^2^	*p*
**(a) Demographics**				
Age in years	14.13 (1.46)	13.82 (1.72)	0.25	0.62
IQ (WAIS-II)	106.25 (15.23)	104.55 (12.24)	0.10	0.76
Years of education	9.50 (1.41)	9.27 (1.49)	0.16	0.69
Age at onset of ADHD (years)	6.56 (2.00)	6.82 (0.98)	0.15	0.70
Social communication questionnaire	9.38 (5.90)	8.46 (4.89)	0.19	0.66
Children’s global assessment scale	51.31 (7.31)	49.73 (8.52)	0.27	0.61
Oppositional defiant disorder comorbidity	6 (38%)	6 (55%)		
**(b) Clinical measures**				
**ADHD-Rating Scale**				
**ADHD-RS total score**	38.13 (8.96)	36.73 (11.22)	0.13	0.72
**ADHD-RS inattention**	20.56 (4.16)	20.46 (4.61)	0.00	0.95
**ADHD-RS hyperactivity/impulsivity**	17.56 (5.63)	16.27 (7.42)	0.26	0.61
***Conner’s Parent Rating Scale (T-score)***				
***ADHD index***	14.68 (3.59)	16.09 (2.91)	1.15	0.29
Global index	85.06 (5.66)	87.18 (6.62)	0.80	0.38
Inattention	83.25 (5.80)	84.64 (6.12)	0.36	0.56
Hyperactivity/impulsivity	86.81 (6.91)	85.36 (10.04)	0.20	0.66
DSM-5 attention	81.31 (6.52)	83.64 (6.62)	0.71	0.41
DSM-5 hyperactivity/impulsivity	87.25 (7.25)	85.09 (10.16)	0.42	0.52
Kiddie-SADS-Present and Lifetime Version (ADHD subscale)				
Total score	14.44 (2.28)	13.73 (3.07)	0.48	0.52
Inattention	7.81 (1.17)	7.64 (1.21)	0.15	0.71
Hyperactivity/impulsivity	6.69 (1.54)	6.09 (2.55)	0.58	0.45
WREMB-R Total score	22.38 (5.83)	21.82 (6.38)	0.06	0.81
Columbia impairment scale	22.25 (10.90)	26.55 (11.81)	0.94	0.34
Side effects	15.94 (6.70)	19.55 (9.02)	1.42	0.25
**(c) Medication status**				
Medication naïve	0 (0%)	1 (9%)		
Off stimulant medication	2 (13%)	3 (27%)		
On stimulant medication	14 (88%)	7 (64%)		
**(d) fMRI-NF runs**				
Number of fMRI-NF runs (max 14)	11.63 (2.53)	12.45 (1.97)	0.83	0.37
Completed 11 + fMRI-NF runs	11 (69%)	8 (73%)	0.05	0.83
Completed all 14 fMRI-NF runs	4 (25%)	6 (55%)	1.14	0.24
Average fMRI-NF performance (%)	57 (8.4)	43 (10.0)	17.479	<0.001

WREMB-R, Weekly Rating of Evening and Morning Behavior-Revised; WASI, Wechsler Abbreviated Score of Intelligence, second edition. SD: Standard deviation.

*Within-group*. Within-group ANOVA comparisons showed a significant decrease in ADHD symptoms in both groups after relative to before fMRI-NF, for most primary and secondary outcome measures (see Table 2 for details). The only exceptions were the ADHD-RS hyperactivity/impulsivity in the rIFC-NF group and the CPRS-R global index in the lPHG-NF group, which only presented a trend-wise reduction (F (df = 1,25) = 4.633, p = 0.057), while the CPRS-R hyperactivity/impulsivity and the CPRS-R DSM-5 hyperactivity/impulsivity did not change significantly in the lPHG-NF group (Table 2).

**Table 2:** Behaviour ratings before and after real-time fMRI neurofeedback training for each ADHD group.

	Pre-fMRI-NF	Post fMRI-NF	Pre-Post	
	Mean (SD)	Mean (SD)	F	*p*
**rIFC-NF group (N = 16)**			F(1,15)	
**ADHD-Rating Scale**				
ADHD-RS total score	38.13 (8.96)	31.60 (11.69)	5.23	**0.037**
ADHD-RS inattention	20.56 (4.16)	17.07 (6.50)	4.969	**0.042**
ADHD-RS hyperactivity/impulsivity	17.56 (5.63)	14.53 (6.36)	4.077	0.062
**Conner’s Parent Rating Scale (T-score)**				
*ADHD index*	14.68 (3.59)	11.00 (5.80)	7.707	**0.014**
Global index	85.06 (5.66)	77.00 (11.94)	8.091	**0.012**
Inattention	83.25 (5.80)	74.13 (8.88)	13.676	**0.002**
Hyperactivity/impulsivity	86.81 (6.91)	79.88 (13.35)	9.779	**0.007**
DSM-5 Inattention	81.31 (6.52)	72.50 (8.61)	7.906	**0.013**
DSM-5 Hyperactivity/impulsivity	87.25 (7.25)	81.75 (12.51)	7.313	**0.016**
**lPHG-NF group (N = 11)**			F(1,10)	
**ADHD-Rating Scale**				
ADHD-RS total score	36.73 (11.22)	28.18 (10.80)	35.958	**<0.001**
ADHD-RS inattention	20.46 (4.61)	15.45 (6.31)	23.667	**0.001**
ADHD-RS hyperactivity/impulsivity	16.27 (7.42)	12.73 (6.23)	11.865	**0.006**
**Conner’s Parent Rating Scale (T-score)**				
*ADHD index*	16.09 (2.91)	11.27 (5.02)	17.066	**0.002**
Global index	87.18 (6.62)	80.91 (13.51)	4.633	0.057
Inattention	84.64 (6.12)	76.64 (11.07)	5.047	**0.048**
Hyperactivity/impulsivity	85.36 (10.04)	81.09 (13.09)	2.325	0.158
DSM-5 Inattention	83.64 (6.62)	70.27 (13.37)	10.306	**0.009**
DSM-5 Hyperactivity/impulsivity	85.09 (10.16)	82.18 (13.22)	1.744	0.216

SD: Standard deviation. In bold: significant difference pre – post training (p < 0.05).

*Between-group*. Between-group repeated measures ANOVA comparisons showed a significant effect of time, with a reduction of all clinical outcome measures (ADHD-RS total scale: F(df = 1,25) = 17.030, p = 0.0001; ADHD-RS Inattention subscale: F(df = 1,25) = 16.773, p = 0.0001; ADHD-RS Hyperactivity/Impulsivity subscale: F(df = 1,25) = 10.659, p = 0.003; CPRS-R ADHD Index: F(df = 1,25) = 20.573, p = 0.0001), but no group or group by time interaction effects.

We also tested for group differences in clinical measures post-treatment. The between-group ANOVA revealed no significant between-group differences in clinical measures after fMRI-NF (Table S1).

### 3.2 Performance in the stop signal task

**Fig. 1:**
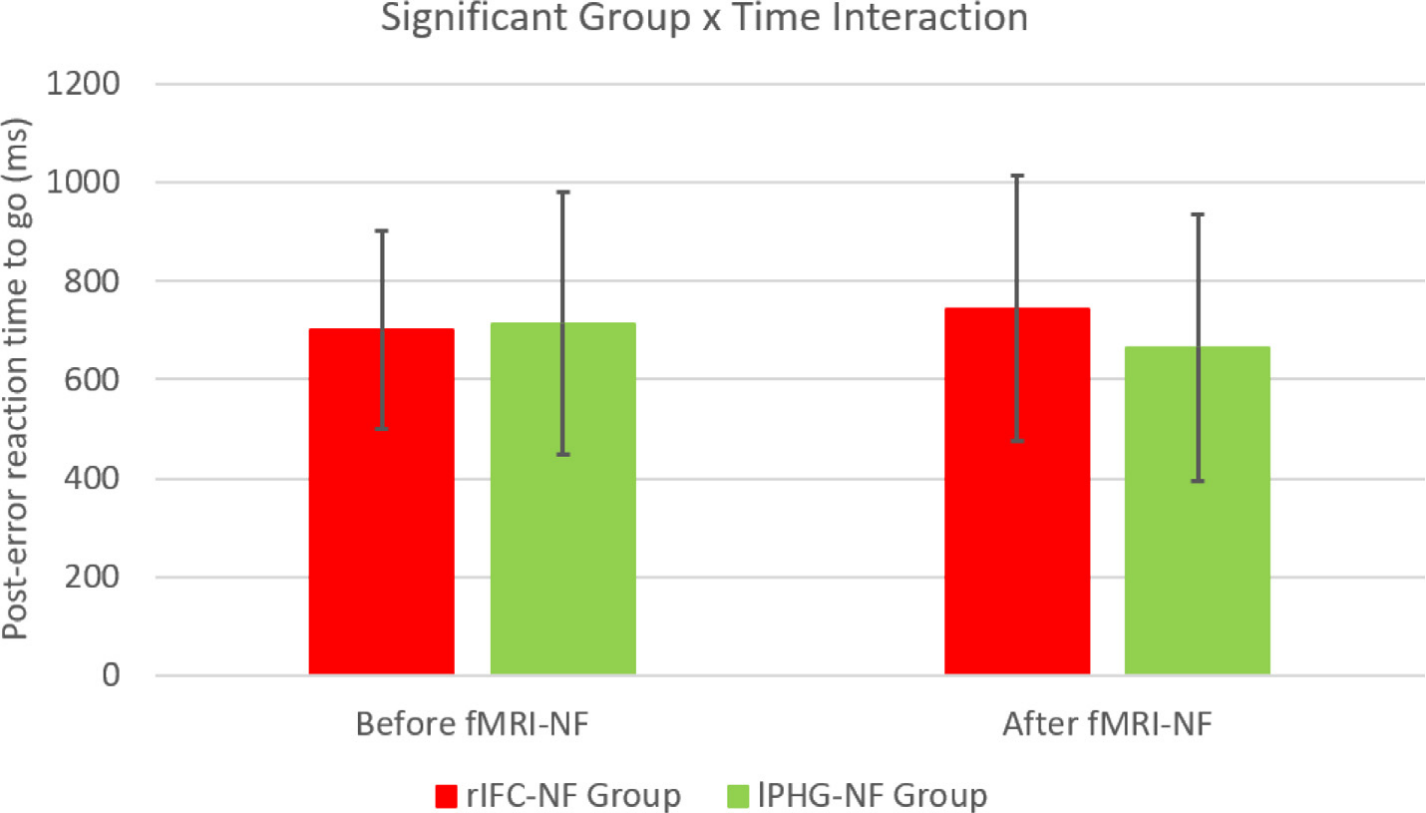
Changes in post-error reaction times to go trials before and after fMRI-NF in the two groups.

Between-group repeated measures ANOVA comparisons showed a significant effect of time, with a reduction of standard deviation of reaction time in both groups (F (df = 1,25) = 8.143, p = 0.009), but there was no effect of group in any dependent variables. The only significant group by time interaction effect was for the post-error reaction time to go signals (F (df = 1,25) = 5.669, p = 0.025), which was increased in the rIFC-NF group relative to the lPHG-NF group post compared to pre fMRI-NF (Table 3, Fig. 1).

**Table 3:** Performance measures in the Stop task at pre- and post-fMRI-NF for each ADHD group.

	Pre-fMRI-NF	Post-fMRI-NF
	**Mean (SD)**	**Mean (SD)**
**(a) rIFC-NF group (N = 16)**		
Stop signal reaction time (ms)	137.81 (183.77)	107.19 (221.05)
Post-error reaction time to go signals (ms)	700.69 (201.25)	744.06 (269.10)
Mean reaction time to go trials (ms)	777.38 (224.99)	803.88 (275.58)
Intrasubject reaction time variability to go trials (ms)	187.81 (48.25)	159.19 (43.67)
Omission errors (%)	5.56 (7.81)	5.13 (5.44)
**(b) lPHG-NF group (N = 11)**		
Stop signal reaction time (ms)	135.36 (107.33)	154.18 (218.00)
Post-error reaction time to go signals (ms)	713.07 (266.56)	664.86 (269.76)
Mean reaction time to go trials (ms)	750.31 (207.13)	756.42 (277.08)
Intrasubject reaction time variability to go trials (ms)	204.51 (100.49)	166.78 (45.45)
Omission errors (%)	10.64 (23.71)	5.82 (11.42)

SD: Standard deviation.

### 3.3 FMRI results

**Fig. 2:**
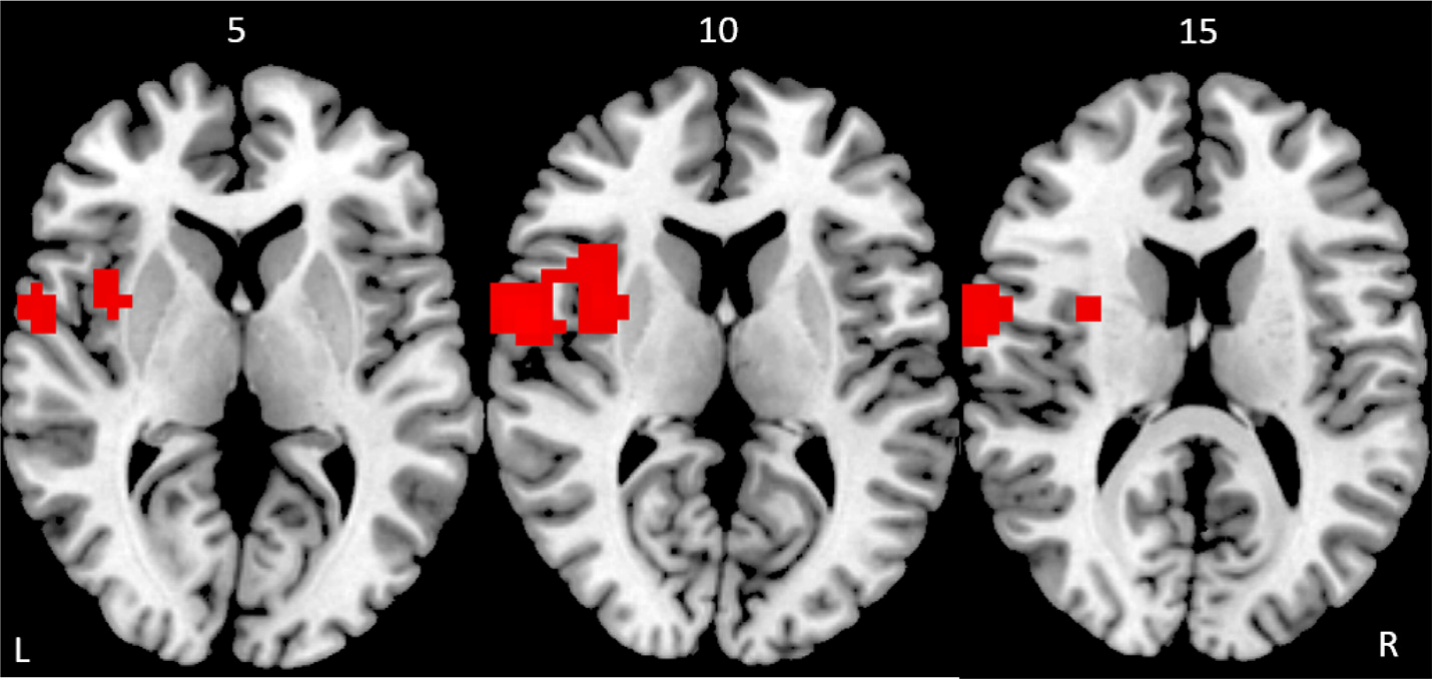
Axial slices showing increased activation in the rIFC-NF group compared to the lPHG-NF group after compared to before fMRI-NF during error monitoring/failed stop trials in the fMRI stop task. Slices shown in mm distance from the anterior-posterior commissure, right side (R) of the image corresponds to the right side of the brain.

The cluster-level p-value was determined in such a way to produce <1 false positive cluster per map (Bullmore et al., 1999b). This was achieved in an ANOVA at voxel-level of p < 0.05 and cluster p < 0.02. In the rIFC-NF group, compared to the lPHG-NF group, the repeated measures ANOVA showed increased activation during the unsuccessful stop–go trials post- relative to pre-fMRI-NF in the left premotor cortex/postcentral gyrus (BA 6/4) and in a cluster comprising the left anterior insula and inferior frontal cortex reaching into putamen (BA 45) (Table 4, Fig. 2). There was no significant increase of activation in the lPHG-NF group compared to the rIFC-NF group.

**Table 4:** Changes in brain activation in the rIFC-NF ADHD group compared to the lPHG ADHD group.

Cluster	Brain regions	Brodmann's Area	Peak Talairach coordinates			Cluster Size (voxels)	Cluster p-value
			x	y	z		
1	Left premotor cortex/postcentral gyrus	6/4	−54	4	7	128	0.008
2	Left inferior frontal/ insula/premotor/ putamen/	44/45/6	−29	4	10	58	0.019

### 3.4 Correlations between clinical and cognitive changes and brain activation changes

**Fig. 3:**
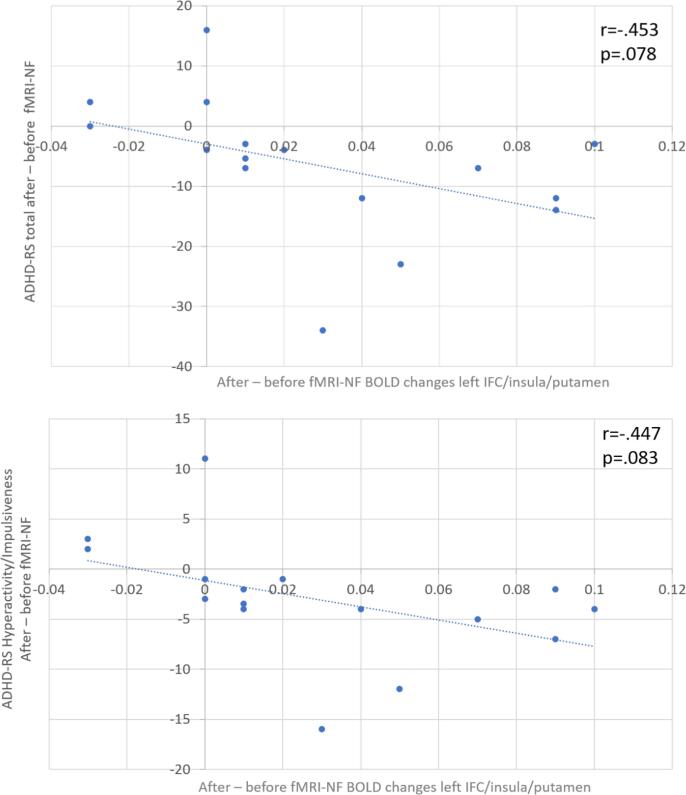
Pearson correlations between brain activation changes in the left IFC/insula/putamen cluster and changes in clinical outcome measures after fMRI-NF of rIFC compared to before fMRI-NF of rIFC.

To test whether the group differences in brain changes to failed stop trials post minus pre fMRI-NF were correlated with NF-induced cognitive or clinical changes in the rIFC-NF group, Pearson correlation analyses were performed in this group. Analyses were undertaken between the post- versus pre- NF brain changes and the corresponding post- versus pre- changes in the primary clinical outcome measures and in the key cognitive measure which is post-error reaction times. For this purpose, the average BOLD response was extracted for each subject in the activation clusters that resulted from the repeated between-group ANOVA analysis. These BOLD responses were then correlated with the changes post- versus pre- fMRI-NF in post-error reaction times to go signals, as well as with the primary clinical outcome measures of the ADHD-RS. There was no significant correlation between the NF-induced changes in brain activation and the NF-induced changes in post-error reaction times. There were two trend-level negative correlations in the left insula/IFC/putamen between the NF-induced changes in brain activation and the NF-induced changes in primary outcome measures (ADHD-RS total: r = −0.453, p = 0.078; ADHD-RS Hyperactivity/impulsivity: r = −0.447, p = 0.083) in the rIFC-NF group (Fig. 3). Once we removed two extreme outliers, values beyond 1.5 times the interquartile range, the correlation became significant for the 14 remaining participants (ADHD-RS total: r = −0.610, p = 0.020; ADHD-RS Hyperactivity/impulsivity: r = −0.600, p = 0.023). This suggests that the increased brain activation in the cluster comprising left inferior fronto-insular-striatal regions in the rIFC-NF relative to the lPHG-NF group after compared to before fMRI-NF was related with improvement in total and in hyperactivity/impulsiveness ADHD symptoms.

### 3.5 Group activation across all 11 fMRI-NF runs in both groups

**Fig. 4:**
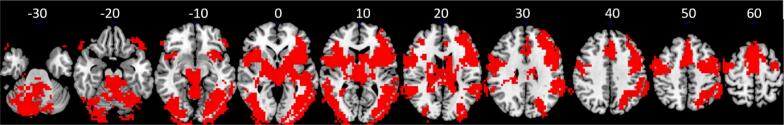
Brain activation in both groups together during fMRI-NF compared to baseline averaged across all 11 runs. Slices shown in mm distance from the anterior-posterior commissure, right side of the image corresponds to the right side of the brain.

We found increased brain activation, averaged across all runs, in both groups in the bilateral inferior frontal lobe/insula, basal ganglia, thalamus, anterior cingulate, SMA, premotor cortex, superior/middle/inferior temporal cortices, right middle frontal, right inferior/superior parietal/precuneus/occipital, the right ventrolateral prefrontal cortex/insula and the left ventrolateral prefrontal/superior temporal cortices (Table 5, Fig. 4).

**Table 5:** Brain activation in both groups combined during Neurofeedback compared to baseline averaged across all 11 runs.

Cluster	Brain regions	Brodmann's Area	Peak Talairach coordinates			Cluster Size (voxels)	Cluster p-value
			x	y	z		
1	Bilateral inferior frontal lobe/insula,basal ganglia, thalamus,anterior cingulate, SMA, premotor, superior/middle/inferior temporal, right middle frontal, right inferior,superior parietal/precuneus, occipital	45/47/44/9/8/34/6	43	15	3	9716	0.0002
2	Right ventrolateral prefrontal/insula	47	51	41	–23	65	0.0004
3	Left ventrolateral prefrontal/superior temporal	47	−47	33	−27	62	0.0004

## 4 Discussion

This study investigated the effects of fMRI-NF of rIFC relative to lPHG on error monitoring performance and underlying brain activation, in adolescents with ADHD. We found slower post-error reaction times to go signals after rIFC fMRI-NF compared to lPHG-NF, suggesting stronger performance adjustment to errors. At the neurofunctional level, fMRI-NF of rIFC compared to that of lPHG was associated with increased activation in error monitoring regions of the left IFC-insular-striatal areas and premotor cortex. This increased activation was trend-wise correlated with rIFC-NF-induced improvements in the primary outcome measures of total ADHD and ADHD hyperactivity/impulsiveness symptoms.

Behaviourally, we found that adolescents with ADHD in the rIFC-fMRI-NF group compared to those in the lPHG-NF group slowed down more after committing errors. In healthy controls, there is typically a performance adjustment after mistakes, reflected by slowing down in the following trials (Danielmeier and Ullsperger, 2011, Schroder and Moser, 2014) and is combination of self-monitoring (error detection/awareness) and adaptive control (behaviour adjustment) (Berwid et al., 2014, Danielmeier and Ullsperger, 2011, Schachar et al., 2004, Shiels and Hawk, 2010, Wiersema et al., 2005). Children and adults with ADHD have poor performance monitoring and typically do not slow down after mistakes (Berwid et al., 2014, Mohamed et al., 2016a, Shiels and Hawk, 2010, Verbruggen and Logan, 2008), observed in a range of tasks such as Go/No-go (Wiersema et al., 2005), choice reaction time (Berwid et al., 2014, Wiersema et al., 2005) and Stop tasks Schachar et al., 2004). The error monitoring deficit seems furthermore independent of the inhibition deficit, and associated with ADHD symptoms (Schachar et al., 2004) and poor self-monitoring and adaptive control (O’Connell et al., 2009, Shiels and Hawk, 2010). FMRI-NF of rIFC therefore appears to have had a positive effect on the error monitoring ability of adolescents with ADHD, making them slow down their reaction times to post-error go trials, suggesting a more carefully adjusting behaviour in response to mistakes.

Interestingly, both groups improved in intrasubject response variability which is one of the most consistently observed impairments in patients with ADHD (Feige et al., 2013, Karalunas et al., 2014, Tamm et al., 2012) and associated with attentional lapses and enhanced DMN activation (Feige et al., 2013, Karalunas et al., 2014, Leth-Steensen et al., 2000, Tamm et al., 2012). Differences in the premotor cortex, insula and temporo-parietal attention areas have been associated with increased response variability in ADHD (Rubia et al., 2007b, Spinelli et al., 2011, Suskauer et al., 2008, Tamm et al., 2012). Stimulant medication furthermore has been shown to induce a reduction, and even a normalisation, in intrasubject response variability in both children (Rubia et al., 2003a, Tamm et al., 2012) and adults with ADHD (Fredriksen et al., 2019). The findings of the current study suggest that fMRI-NF training - independent of the region being trained – improves intrasubject response variability, which may reflect a generic effect of fMRI-NF in improving attention control, given that it was observed in both groups. In fact, the group activation map across all subjects showed increased activation during NF across all 11 runs in areas of the ventral attention network including bilateral inferior frontal cortex, striato-thalamic and right inferior parietal regions (Table 5, Fig. 4). However, the findings should be taken with caution, as the improvement of intrasubject variability in both groups could potentially also be attributed to a generic practice effect, not related to fMRI-NF.

The improvement in post-error performance adjustment was paralleled at the brain level, where rIFC fMRI-NF relative to lPHG fMRI-NF resulted in increased activation in adolescents with ADHD in error monitoring regions of the left IFC, anterior insula, putamen and premotor cortex. Although there is some overlap between inhibition and error monitoring networks, the right IFC has most consistently been associated with motor response inhibition (Aron et al., 2004, Rubia et al., 2003b) while the left IFC, along with insula, premotor cortex and putamen, is a key region of error monitoring (Mohamed et al., 2016b, Swick and Turken, 2002, Zhang and Li, 2012). These regions are typically under-activated in children and adults with ADHD relative to healthy controls during failed stop trials (in addition to other region such as left DLPFC, parietal and temporal cortices, posterior cingulate (PCC)/precuneus, and thalamus) (Rubia et al., 2005, Rubia et al., 2011a). The anterior insula in particular is a key area that has also been found to be under-activated in the context of inhibition in ADHD in a recent meta-analysis of fMRI studies of cognitive control, where it was also smaller in structure (Norman et al., 2016). The anterior insula is particular related to error awareness (Ullsperger and von Cramon, 2004) which is thought to influence post-error adjustment, especially during the stop task (Shiels and Hawk, 2010), as part of adaptive control (Klein et al., 2007). ADHD has been associated with poor error awareness (O’Connell et al., 2009) and under-activation of the mediating insular region (Klein et al., 2013). It has even been hypothesised that the self-regulation impairment in ADHD could be due to impaired error awareness (Shiels and Hawk, 2010).

Both medial and lateral prefrontal cortices have been implicated in error monitoring, but their roles appear to be related to different components (Cavanagh et al., 2009). The medial prefrontal cortex (ACC) seems to be responsible for action monitoring and serves as an alarm. On the other hand, the lateral prefrontal cortex (IFC) seems to be in charge of the cognitive control aspect by reallocating attentional resources and increasing the motor threshold (Botvinick et al., 2001, Cavanagh et al., 2009, Eichele et al., 2008), it appears to implement the behaviour adjustment following an error (Ridderinkhof et al., 2004). Both components of error monitoring have been shown to be impaired in children with ADHD (Rubia et al., 2005, Rubia et al., 2011b, Shiels and Hawk, 2010). Our findings of NF-induced upregulation of left IFC activation could therefore suggest that fMRI-NF of rIFC restores the cognitive control aspect of error monitoring, rather than the action monitoring aspects, as we found no effect on ACC activation.

The premotor cortex, connected to the anterior insula via the pre-supplementary motor area, has also been associated with error detection (Bastin et al., 2017, Desmurget and Sirigu, 2009) to help evaluating consequences and adapting future actions (Pardo-Vazquez et al., 2009). In ADHD, the premotor cortex has been shown to be under-activated during error monitoring together with other regions (see above) (Rubia et al., 2011b). Furthermore, premotor under-activation has been found to be associated with post-error slowing (Rubia et al., 2011b) and intrasubject response variability in ADHD (Spinelli et al., 2011, Suskauer et al., 2008, Tamm et al., 2012).

Taken together, our results show that fronto-insular-striatal and premotor activation related to the cognitive control aspects of error monitoring in ADHD, which are typically been found to be underactivated relative to healthy controls (Rubia et al., 2005, Rubia et al., 2011a), are increased by rIFC fMRI-NF.

We have previously shown that, relative to placebo, a single dose of MPH upregulated and normalised activation in ADHD adolescents during error monitoring in the anterior insula, left IFC, premotor cortex and striatal regions, as well as other posterior attention regions (Rubia et al., 2011b). The findings presented here thus suggest that fMRI-NF of the rIFC may have similar effects to MPH in increasing activation of these IFC, insular and premotor regions, but without the medication-associated side effects.

The finding of increased left fronto-striatal activation during error monitoring with fMRI-NF suggest that self-regulation of an isolated prefrontal region has a more widespread effect on other fronto-striatal systems in ADHD. During inhibition, we previously found increased NF-induced activation in the right IFC as well as in parietal regions in the rIFC-NF compared to the lPHG-NF group (Alegria et al., 2017). The increased activation in left fronto-insular-striatal and premotor brain networks related to error monitoring reported here, suggests that fMRI-NF not only increases activation in the targeted upregulated region, i.e. rIFC, but also in wider left homologue regions mediating associated self-control and self-monitoring functions. This more widespread effect of fMRI-NF of rIFC on other brain systems extends our functional connectivity findings in patients with ADHD. Indeed, we showed that the upregulation of rIFC was associated with increased functional connectivity in a cognitive control network, along with decreased functional connectivity with areas of the default mode network (Rubia et al., 2019). Together, these findings are of great importance to the application of fMRI-NF as an alternative treatment for ADHD, as they show that fMRI-NF of the rIFC has an impact on wider left and right fronto-striato-insular networks of self-regulation in adolescents with ADHD.

Regions that are crucial for performance monitoring may be the same regions that are important for self-regulation. Indeed, a meta-analysis of fMRI-NF studies (Emmert et al., 2016), showed that rIFC, anterior insula, premotor cortex and striatal areas, along with DLPFC and ACC, were consistently activated during self-regulation, independent of the upregulated region. Greater self-regulation was also associated with increased activation of the fronto-cingular-striatal cognitive control network, one of the three regulation networks in fMRI-NF (Sitaram et al., 2017). Interestingly, in this sample of ADHD adolescents, we found that fMRI-Neurofeedback activated a strikingly similar network of self-control, including regions of bilateral inferior frontal cortex/insula, anterior cingulate, SMA, premotor and striato-thalamic regions as the network observed in healthy adults in the meta-analysis of Emmert and collaborators in 2016 (Table 5, Fig. 4). The findings suggest that ADHD patients activate the same fronto-cingulo-striato-thalamic self-regulation networks when they undergo fMRI-NF as healthy adults do.

The changes in error-monitoring activation induced by fMRI-NF were negatively correlated with positive changes in ADHD symptoms (which became significant once we removed extreme outliers), suggesting that larger increases of brain activation in left fronto-insular-striatal error monitoring regions were associated with greater improvement in ADHD symptoms. It has been found previously that the neural correlates of error monitoring correlated with severity of hyperactivity and impulsivity symptoms in adult ADHD (Balogh et al., 2017). In our proof-of-concept study, the up-regulation of the rIFC during the NF training as well as the changes in functional connectivity induced by rIFC-NF were correlated with ADHD symptom improvements after fMRI-NF training (Alegria et al., 2017, Rubia et al., 2019). It is thus reassuring that error-monitoring associated brain changes induced by rIFC-NF in adolescents with ADHD seem to be associated with clinical improvements.

Although the implications of these brain-behaviour correlations are very promising with respect to the potential clinical future use of fMRI-NF, these latter results must be considered with caution as they did not reach statistical significance. We furthermore found no correlation between the changes in post-error slowing and brain activation changes, which would have further strengthened the association. Our sample size was relatively small and our study was not powered to show such associations, so further studies will be required in order to determine whether these results are valid and replicable.

Notably, both groups showed improvement of ADHD symptoms, while the brain activation and performance effects were specific to the rIFC-NF group. This is similar to what we found in our previously study where only the rIFC-NF group had increased rIFC and parietal activation during inhibition in the fMRI stop task and improved in sustained attention (Alegria et al., 2017). Since the fMRI tasks were specifically selected to be improved by rIFC upregulation, more benefits were expected for rIFC than lPHG self-regulation. Possibly, we might have seen improvements specific to the lPHG-NF group had we included an episodic memory or visuo-spatial processing task, mediated by lPHG (Aminoff et al., 2013). The shared clinical improvement may be due to both groups learning to self-regulate their brain, even in different regions. In fact, this is supported by our whole brain group analysis where we showed that both groups during neurofeedback activated a strong cognitive control network of self-regulation, including bilateral inferior frontal cortex, insula, anterior cingulate/SMA, and striato-thalamic regions (Table 5, Fig. 4). In our published study, we also found that there was progressively increased activation with increasing number of NF runs in both groups in dorsolateral and parietal attention networks independent of the region the two groups had to self-regulate (see Alegria et al., 2017, Fig. 5A). It is also possible that fMRI-NF was more difficult for the lPHG-NF group and this could have resulted in clinical improvement in the control group. Indeed, it has been found that anterior, higher-level association regions are easier to self-regulate than posterior and smaller regions. For example, healthy adults could successfully learn to upregulate the anterior insula, while they could not modulate the middle parahippocampal region (Lawrence et al., 2014). Similarly, anterior cingulate could be successfully upregulated as opposed to posterior cingulate (Guan et al., 2015) and higher visual-association areas were more easily self-regulated than primary visual regions (Harmelech et al., 2015). In our data, this is also supported by better fMRI-NF performance during the rocket game in the rIFC group than in the lPHG group. It is hence possible that the lPHG-NF group had to work harder to self-regulate the lPHG compared to the rIFC group and this could have led to similar clinical improvements. While error performance networks, that are specifically mediated by prefrontal regions, benefitted more from the rIFC self-regulation.

A key limitation of this study is the small sample size, which was underpowered for performance and brain-behaviour correlation analyses, as discussed above. Because of the small sample size and because they were exploratory, the behavioural analyses were not corrected for multiple comparisons. The fMRI findings *per se* were stronger than the behavioural findings, in line with evidence that fMRI is more sensitive in detecting treatment effects in ADHD than behavioural or clinical data (Cubillo et al., 2014, Rubia et al., 2011b, Smith et al., 2013). Another limitation is related to ADHD medication. Although there was no significant difference between the groups in medication status or medication type, they were not identical, and this could have influenced the findings. Also, since most of the patients (70%) were taking stimulant medication, the effects of fMRI-NF need to be seen in this context. We do not know whether the effects of fMRI-NF would be different in entirely medication-naïve ADHD patients. Indeed, we know that stimulant medication improves attention, self-control, and error monitoring and increases the activation of the rIFC and other fronto-striatal and cingulate error monitoring regions during stop task performance (Bédard et al., 2015, Coghill et al., 2014, Fredriksen et al., 2019, Hammerness et al., 2014, Rubia et al., 2011b, Rubia et al., 2014b). It is hence possible that a group of ADHD patients who are 100% medication-naïve may find it more difficult to self-regulate their brain activation than medicated patients. Future studies should investigate whether fMRI-NF has differential effects in patients with ADHD with and without stimulant medication. Finally, as mentioned above, given that the lPHG was used as control region, it would have been interesting to test whether the control group improved in tasks that measure functions mediated by lPHG such as episodic memory or visuospatial processing (Aminoff et al., 2013).

## 5 Conclusion

In conclusion, the findings of the current study show that fMRI-NF of the rIFC compared to fMRI-NF of a posterior control region had a positive effect on behavioural and neurofunctional correlates of error monitoring in ADHD. fMRI-NF induced increased activation of left-hemispheric fronto-insular-striatal self-monitoring regions, and also resulted in a slowing down of response times after mistakes, suggesting better post-error reaction time adjustments. Furthermore, these change in brain activation were correlated with improvements in ADHD symptoms, albeit only at a trend level of statistical significance. The upregulation of the performance monitoring network after fMRI-NF was similar to the effects of ADHD medication previously observed on the same regions, but with the advantage that fMRI-NF has no known side effects. The findings highlight the importance of the wider regional effects that fMRI-NF of a particular self-control target region has on other self-regulatory networks, such as, in this case, those typically impaired in ADHD; this makes fMRI-NF a promising potential neurotherapy for ADHD (Rubia, 2018).

## Funding

This work, AA and MW were supported by a grant from Action Medial Research (grant number: 1890) to KR. Additional support was provided by the National Institute for Health Research (NIHR) Biomedical Research Centre at South London and the Maudsley NHS Foundation Trust and King’s College London and by the Medical Research Council (MRC) (MR/P012647/1) to KR which also supported MC. AA was supported by a Ph.D studentship from the Institute of Psychiatry, Psychology and Neuroscience, King’s College London. The funders had no involvement in the collection, analysis and interpretation of data; in the writing of the report; or in the decision to submit the article for publication.

## CRediT authorship contribution statement

**M. Criaud:** Formal analysis, Writing - original draft, Writing - review & editing, Visualization. **M. Wulff:** Investigation. **A.A. Alegria:** Investigation. **G.J. Barker:** Methodology, Software, Validation, Resources. **V. Giampietro:** Methodology, Software, Validation, Resources. **K. Rubia:** Conceptualization, Formal analysis, Resources, Data curation, Writing - review & editing, Supervision, Project administration, Funding acquisition.
