# Supplementary material for: Increased left inferior fronto-striatal activation during error monitoring after fMRI neurofeedback of right inferior frontal cortex in adolescents with attention deficit hyperactivity disorder
Source: Neuroimage Clin. 2020 Jun 10;27:102311. doi: 10.1016/j.nicl.2020.102311 (PMC7306625; doi:10.1016/j.nicl.2020.102311)
Supplement: Supplementary data 1 [file mmc1.docx]

**Increased left inferior fronto-striatal activation during error monitoring after fMRI neurofeedback of right inferior frontal cortex in adolescents with Attention Deficit/ Hyperactivity Disorder**

**Supplementary material**

**S1. Materials and methods**

*S1.1. FMRI Data Acquisition and Processing*

All participants who completed the stop task (16 participants in the rIFC-NF group and 11 in the lPHG-NF group) were included in the analyses. Functional and structural data were acquired on a 3T GE (General Electric) MR750 MRI scanner with a 12-channel head coil. The body coil was used for RF transmission. At the beginning of each scanning session, a T1-weighted structural scan (repetition time (TR)/echo time (TE) = 7.312/3.016 ms, flip angle = 11 degrees, 196 x 1.2 mm slices, matrix size 256 x 256, 27 cm FOV, voxel size = 1.05 x 1.05 x 1.2 mm3) was collected and used as structural localizer. FMRI-NF scans were collected using a T2*-weighted gradient echo, echo-planar image (EPI) sequence (TR/TE = 2000/30 ms, flip angle = 75°, 40 x 3 mm slices with a 0.3 mm slice gap, matrix size 64 x 64, 21.1 cm FOV, voxel size = 3.3 x 3.3 x 3.3 mm3). A whole-brain higher resolution gradient echo, EPI scan was also acquired in the inter-commissural plane (TR/TE = 3.000/30 ms, flip angle = 90°, 43 slices, slice thickness = 3.0 mm with a 0.3 mm slice gap, matrix size 128 x 128, 21.1 cm FOV, voxel size = 1.65 x 1.65 x 3.3 mm^3^) for standard space normalization of individual activation maps.

A custom fMRI-NF interface system (Bodurka and Bandettini, 2008) and the AFNI software (Cox, 1996) were used for real-time transfer and analysis of the fMRI data. The fMRI-NF interface system ran on the scanner hardware to access the fMRI scans as they were reconstructed. The images were then transferred to a Linux server to be pre-processed using the real-time options of AFNI. The effects of head motion were corrected in real-time by the AFNI software. We used the CA_N27_ML/TT_N template to define the target ROIs (ROI_TAR_: rIFC; or lPHG) in AFNI structurally for each adolescent before each fMRI-NF session. The ROI of rIFC included the pars triangularis (14,138 voxels in the Talairach space of the template and 385 voxels when reversed-mapped to fMRI space) and the pars orbitalis (11,484 voxels in the Talairach space of the template and 308 voxels when reversed-mapped to fMRI space). The ROI of lPHG contained 5,976 voxels in the Talairach space of the template and 149 voxels when reversed-mapped to fMRI space. A customized AFNI script created a mask of the two target ROIs. A mask of the white matter was also created to use as reference ROI (ROI_REF_) to cancel out non-specific global brain effect. The masks, defined in Talairach space, were mapped on the T1-weighted structural image and then reversed-mapped onto a two-volume EPI localizer image that was used to register the fMRI images to (across all training runs within a visit).

The mean BOLD signal was extracted from each ROI by applying the masks to the pre-processed fMRI data. For each new brain volume (e.g. every TR/two seconds), AFNI calculated a new set of values for each ROIs. These values were transferred to an in-house program that generated the feedback by displaying a moving rocket. The neurofeedback signal was calculated as:

((ROI_TAR_-ROI_REF_) – (ROI_TAR_Previous – ROI_REF_Previous))

where ROI_TAR_Previous and ROI_REF_Previous are the average activation of rIFC or lPHG, and of white matter, respectively, during the previous rest block. In other word, the NF signal was a function of the difference current ROI_TAR_ activity (averaged over 3 TR periods, in order to reduce jitter) to the averaged activity of the previous rest block. All values were being measured relative to the corresponding white matter signal, that represents global signal of no interest. Before each fMRI-NF run, participants were informed/reminded of the delay (~6s) in the feedback they would receive, caused by both hemodynamic delay and data processing time.

*S1.2. FMRI Stop task Data Acquisition*

Each participant underwent the fMRI Stop task before the first and after the last fMRI-NF run. Thirty-eight interleaved slices covering the whole brain were acquired parallel to the anterior-posterior commissure. 200 T2*-weighted volumes were collected with TR/TE = 1.800/3 ms, flip angle = 75°, matrix size 64 x 64, 21.1 cm FOV, voxel size = 3.3 x 3.3 x 3.3 mm3.

*S1.3. Data Analyses of the fMRI Stop Task*

The individual and group-level analysis methods are described in detail elsewhere (Brammer et al., 1997; Bullmore et al., 1999a, 1999b). Version 4.1 of the non-parametric XBAM software package (Brammer et al., 1997) was used to analyse the data.

fMRI data were first processed to minimize motion related artefacts (Bullmore et al., 1999a). A 3D volume consisting of the average intensity at each voxel over the whole experiment was calculated and used as a template. The 3D image volume at each time point was then realigned to this template by computing the combination of rotations (around the x, y, and z axes) and translations (in x, y, and z) that maximized the correlation between the image intensities of the volume in question and the template (rigid body registration). After realignment, data were then smoothed using a Gaussian filter at 7.8 mm FWHM (full-width half-maximum) to improve the signal-to-noise ratio of the images. After motion correction, smoothing, global detrending and spin-excitation history correction, time series analysis of individual data was performed with a wavelet-based data resampling method (Bullmore et al., 2001, 1999b).

*Individual Analysis.* The main experimental condition of error monitoring in the stop task, e.g. failed stop trials, against an implicit baseline, e.g. the go trials, was obtained using a standard GLM approach. The two conditions were convolved with 2 Poisson model functions (peaking at 4 and 8 s). The weighted sum of these convolutions giving the best fit (least-squares) to the time series at each voxel was calculated. A goodness-of-fit statistic (SSQ ratio) was then computed at each voxel consisting of the ratio of the sum of squares of deviations from the mean intensity value due to the model (fitted time series) divided by that of the squares due to the residuals (original minus model time series). The appropriate null distribution for assessing significance of any given SSQ-ratio was established using a wavelet-based data resampling method (Bullmore et al., 2001) and applying the model-fitting process to the resampled data. This process was repeated 20 times at each voxel, resulting in 20 null parametric maps of SSQ-ratio for each subject, which were combined to give the overall null distribution of SSQ-ratio. The same permutation strategy was applied at each voxel to preserve the spatial correlation structure of the data. Individual SSQ-ratio maps were then transformed into standard space, first by rigid body transformation of the fMRI data into a high-resolution EPI image of the same subject, and then by affine transformation onto a Talairach template (Talairach and Tournoux, 1988).

*Group Analysis*. Two group activation maps (pre- and post-fMRI-NF) for each NF group were produced for the experimental condition by calculating the median observed SSQ-ratio across all subjects at each voxel in standard space and testing them against the null distribution of median SSQ-ratios computed from the identically transformed wavelet re-sampled data (Brammer et al., 1997; Bullmore et al., 2001). A repeated-measures ANOVA was then conducted to test the interaction of time (pre, post) by group (rIFC-NF group, lPHG-NF group). The voxel-level threshold was first set to p < 0.05 to give maximum sensitivity and to minimize type II errors. After that, a cluster-level threshold was computed for the resulting 3D voxel clusters and set in such a way as to produce less than one false positive cluster per map. The necessary combination of voxel- and cluster-level thresholds was not assumed from theory but rather was determined by direct permutation, giving excellent type I error control (Bullmore et al., 1999b). Cluster mass rather than a cluster-extent threshold was used to minimize discrimination against possible small, but strongly responding, foci of activation (Bullmore et al., 1999b).

**TableS1 :** Clinical differences after fMRI-NF.

|  | **rIFC-NF group (N=16)** | **lPHG-NF group (N=11)** | **Between-subject ANOVA** | |
| --- | --- | --- | --- | --- |
|  | **Mean (SD) or n (%)** | **Mean (SD) or n (%)** | **F (1,25)/χ^2^** | ***p*** |
| **ADHD-Rating Scale** |  |  |  |  |
| **ADHD-RS total score** | 31.60 (11.30) | 29.20 (10.81) | 0.622 | 0.438 |
| **ADHD-RS inattention** | 17.07 (6.28) | 16.00 (6.38) | 0.449 | 0.509 |
| **ADHD-RS hyperactivity/impulsivity** | 14.53 (6.14) | 13.20 (6.36) | 0.540 | 0.469 |
| ***Conner’s Parent Rating Scale (T-score)*** |  |  |  |  |
| ***ADHD index*** | 11.00 (5.80) | 11.40 (5,28) | 0.016 | 0.900 |
| Global index | 76.98 (11.92) | 82.00 (13.72) | 0.649 | 0.428 |
| Inattention | 74.09 (8.90) | 77.40 (11.36) | 0.437 | 0.515 |
| Hyperactivity/impulsivity | 79.87 (13.40) | 82.00 (14.07) | 0.051 | 0.824 |
| DSM-5 attention | 72.53 (8.61) | 71.50 (13.72) | 0.289 | 0.596 |
| DSM-5 hyperactivity/impulsivity | 81.76 (12.48) | 82.60 (13.86) | 0.006 | 0.939 |
| WREMB-R Total score | 21.93 (5.70) | 17.80 (6.46) | 3.220 | 0.085 |
| Columbia impairment scale | 21.69 (7.54) | 22.50 (11.39) | 0.053 | 0820 |
| Side effects | 15.77 (6.14) | 14.89 (8.50) | 0.104 | 0.750 |

WREMB-R, Weekly Rating of Evening and Morning Behavior-Revised; WASI, Wechsler Abbreviated Score of Intelligence, second edition. SD: Standard deviation.
